# Supplementary material for: Biocompatibility and antimicrobial efficacy of iodine-supported titania nanotubes on 3D-printed Ti-6Al-4V implants
Source: PLoS One. 2025 Dec 26;20(12):e0339618. doi: 10.1371/journal.pone.0339618 (PMC12742766; doi:10.1371/journal.pone.0339618)
Supplement: S1 Fig — (PDF) [file pone.0339618.s001.pdf]

Supporting information

Supplement 1. The calibration curve for iodine by ICP/MS. Each point represents the average of three readings.

Iodine-127 calibration curve

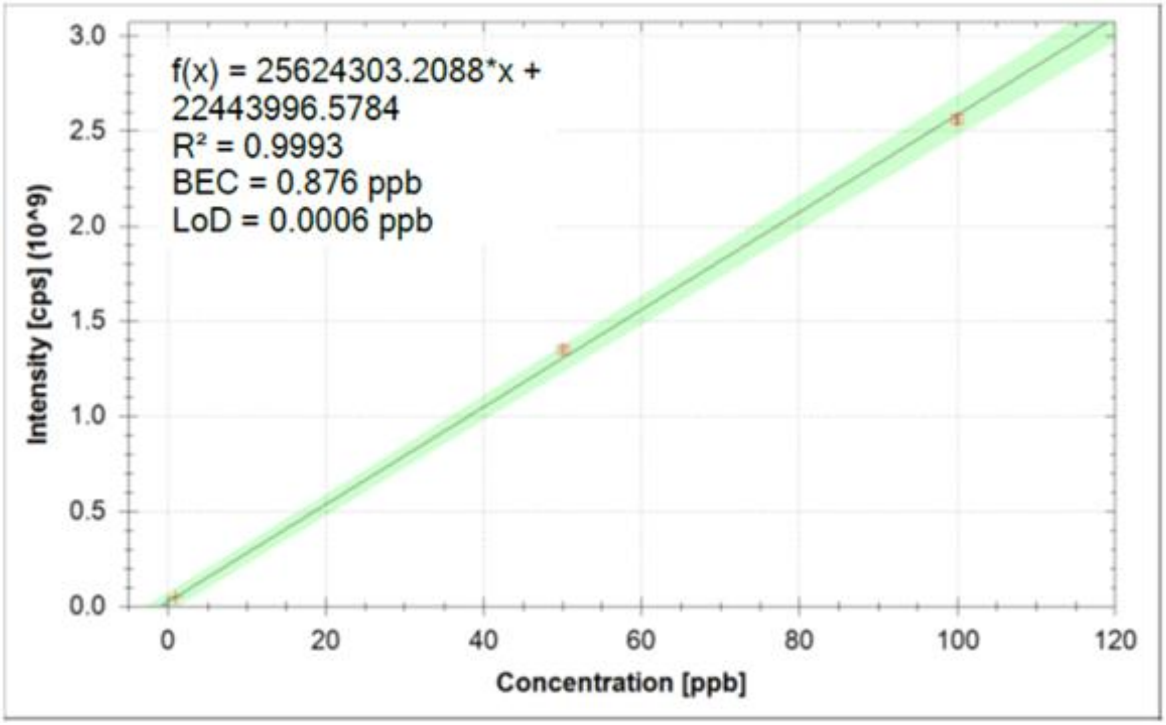

Potassium iodide (Sigma Aldrich, USA) was used for the preparation of the calibration curve. The samples were diluted using a calibration blank of 1% (v/v) HNO_3. The calibration standard solutions of 5, 50, and 100 ppb were used for the calibration to analyse I-content in the I-TNTs specimens. The calibration curves shown in supplement 1 demonstrate excellent linearity over the evaluated concentration range, with correlation coefficients (R) of the calibration curves of 0.9993. For sample dilutions, initial and final volumes were entered into the MassHunter software to enable automated calculation of dilution factors and resultant final sample concentrations**.**
