# Supplementary material for: Biocompatibility and antimicrobial efficacy of iodine-supported titania nanotubes on 3D-printed Ti-6Al-4V implants
Source: PLoS One. 2025 Dec 26;20(12):e0339618. doi: 10.1371/journal.pone.0339618 (PMC12742766; doi:10.1371/journal.pone.0339618)

Supporting information

Supplement 2. Relative cell viability (%) of Saos-2 cells after being treated with a positive control (SLS).

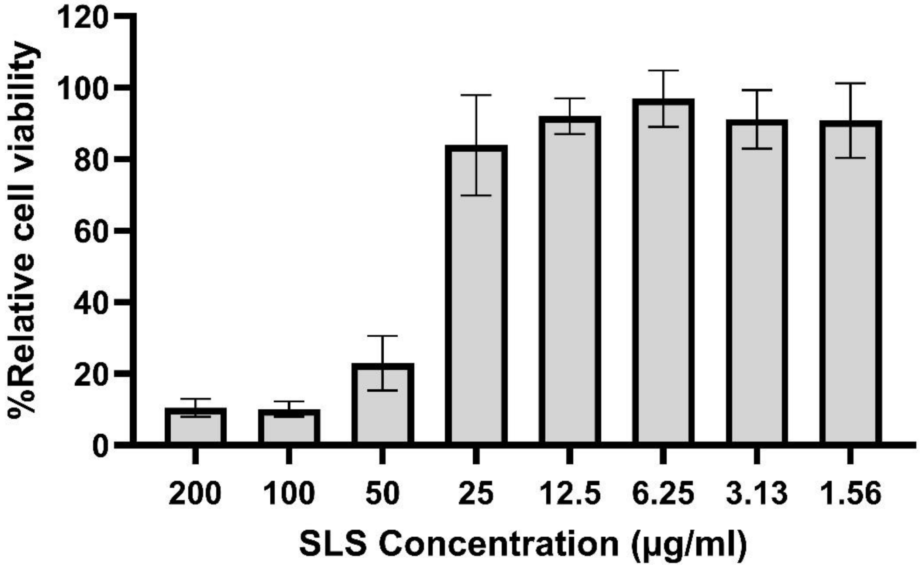

Supplement: S2 Fig — (PDF) [file pone.0339618.s002.pdf]
