# Supplementary material for: Biocompatibility and antimicrobial efficacy of iodine-supported titania nanotubes on 3D-printed Ti-6Al-4V implants
Source: PLoS One. 2025 Dec 26;20(12):e0339618. doi: 10.1371/journal.pone.0339618 (PMC12742766; doi:10.1371/journal.pone.0339618)
Supplement: S3 File — (PDF) [file pone.0339618.s003.pdf]

Supporting information

Supplement 3. Fitting analysis of kinetics models for cumulative iodine release profile

The preliminary fitting analysis was performed using the following sequential steps:

1. As previously stated, a limitation of this study was the quantification of the total iodine content directly on the implant surface. Consequently, we relied on the theoretical iodine loading within the titania nanotubes to define 100% release. To achieve this, the total theoretical iodine content of the I-TNT samples was first calculated. Based on the established formulation [1], the theoretical iodine content was 0.038 wt.-%. Using the average weight of the I-TNT samples (768.53 ± 20.71 mg (detailed in the *minimal data set*), the total theoretical iodine content was calculated to be

$$\frac{0.038x768.53x1000}{100} = 292.04\ \mu g$$

2. The percentage of cumulative iodine release at each sampling point was subsequently calculated by normalizing the measured iodine content to the total theoretical iodine content.

3. To identify the release kinetics, the experimental data were fitted to several key kinetic models, including the Zero-order, First-order, Higuchi, Hixson-Crowell, and Korsmeyer–Peppas models. The best-fit model was determined by comparing the correlation coefficient (R^2) values. This fitting analysis was performed using Microsoft Excel.

The table below shows the x-axis and y-axis for the linear plot of each kinetic model.

Kinetic model	Y-axis	X-axis
First-order	log cumulative % drug remaining	time (hrs)
Zero-order	cumulative % drug released	time (hrs)
Higuchi	cumulative % drug released	square root of time
Hixson-Crowell	cube root of the percentage of the drug remaining	time (hrs)
Korsmeyer-Peppas	log cumulative % drug released	log of time

3.1 Fitting analysis of kinetics models for I-TNT A1

Time (Hr)	Cumulative % drug released	% drug remaining	Square root time	log Cumu % drug remaining	log time	log Cumu % drug released	Cube Root of % drug Remaining(Wt)	Wo-Wt
1	0.337	99.663	1.00	1.999	0.000	0.000	4.636	0.000
12	0.506	99.494	3.46	1.998	1.079	-0.296	4.634	0.008
24	0.926	99.074	4.9	1.996	1.380	-0.033	4.627	0.015
168	11.764	88.236	12.96	1.946	2.225	1.071	4.452	0.190
336	13.72	86.28	18.33	1.936	2.526	1.137	4.419	0.223
672	14.587	85.413	25.92	1.932	2.827	1.164	4.404	0.238

**Kinetic model**	**Linear plot**	**Correlation Coefficient (R^2)**
First-order	y = -0.0001x + 1.9893	0.738
Zero-order	y = 0.0229x + 2.3524	0.731
**Higuchi**	y = 0.6842x – 0.6188	**0.892**
Hixson-Crowell	y = 0.0004x + 0.0363	0.735
Korsmeyer-Peppas	y = 0.4274x – 0.1386	0.717

3.2 Fitting analysis of kinetics models for I-TNT A5

Time (Hr)	Cumulative % drug released	% drug remaining	Square root time	log Cumu % drug remaining	log time	log Cumu % drug released	Cube Root of % drug Remaining (Wt)	Wo-Wt
1	0.374	99.626	1.000	1.998	0.000	0.000	4.636	0.000
12	0.529	99.471	3.464	1.998	1.079	-0.276	4.633	0.009
24	1.06	98.94	4.899	1.995	1.380	0.025	4.625	0.017
168	12.712	87.288	12.961	1.941	2.225	1.104	4.436	0.206
336	14.594	85.406	18.330	1.931	2.526	1.164	4.404	0.238
672	15.45	84.55	25.923	1.927	2.827	1.189	4.389	0.253

Kinetic model	Linear plot	Correlation Coefficient (R^2)
First-order	y = -0.0001x + 1.9883	0.729
Zero-order	y = 0.0242x + 2.5685	0.722
**Higuchi**	y = 0.7253x – 0.5951	**0.886**
Hixson-Crowell	y = 0.0004x + 0.0397	0.726
Korsmeyer-Peppas	y = 0.4393x – 0.1337	0.739

3.3 Fitting analysis of kinetics models for I-TNT B1

Time (Hr)	Cumulative % drug released	% drug remaining	Square root time	log Cumu % drug remaining	log time	log Cumu % drug released	Cube Root of % drug Remaining (Wt)	Wo-Wt
1	0.302	99.698	1.00	1.999	0.000	0.000	4.637	0.000
12	0.455	99.545	3.46	1.998	1.079	-0.342	4.635	0.007
24	0.875	99.125	4.9	1.996	1.380	-0.058	4.628	0.014
168	10.855	89.145	12.96	1.950	2.225	1.036	4.467	0.175
336	12.629	87.371	18.33	1.941	2.526	1.101	4.437	0.205
672	13.346	86.654	25.92	1.938	2.827	1.125	4.425	0.217

Kinetic model	Linear plot	Correlation Coefficient (R^2)
First-order	y = -0.0001x + 1.9901	0.732
Zero-order	y = 0.0209x + 2.1797	0.726
**Higuchi**	y = 0.6274x – 0.5516	**0.889**
Hixson-Crowell	y = 0.0003x + 0.0336	0.729
Korsmeyer-Peppas	y = 0.4131x – 0.1427	0.692

3.4 Fitting analysis of kinetics models for I-TNT B5

Time (Hr)	Cumulative % drug released	% drug remaining	Square root time	log Cumu % drug remaining	log time	log Cumu % drug released	Cube Root of % drug Remaining(Wt)	Wo-Wt
1	0.319	99.681	1.00	1.999	0.000	0.000	4.637	0.000
12	0.489	99.511	3.46	1.998	1.079	-0.311	4.634	0.008
24	0.904	99.096	4.9	1.996	1.380	-0.044	4.628	0.014
168	11.318	88.682	12.96	1.948	2.225	1.054	4.459	0.183
336	12.855	87.145	18.33	1.940	2.526	1.109	4.434	0.208
672	13.995	86.005	25.92	1.935	2.827	1.146	4.414	0.228

Kinetic model	Linear plot	Correlation Coefficient (R^2)
First-order	y = -0.0001x + 1.9899	0.742
Zero-order	y = 0.0218x + 2.2392	0.731
**Higuchi**	y = 0.6517x – 0.5847	**0.894**
Hixson-Crowell	y = 0.0004x + 0.0345	0.738
Korsmeyer-Peppas	y = 0.4192x – 0.1394	0.708
