# Supplementary material for: Biocompatibility and antimicrobial efficacy of iodine-supported titania nanotubes on 3D-printed Ti-6Al-4V implants
Source: PLoS One. 2025 Dec 26;20(12):e0339618. doi: 10.1371/journal.pone.0339618 (PMC12742766; doi:10.1371/journal.pone.0339618)
Supplement: Attachment — Submitted filename: Response to Reviewers comments.docx [file pone.0339618.s005.docx]

**Response to Editor's Comments**

**Q. No. 1	It will be helpful to include EDX/ XPS or PIXE elemental analysis data for validation of concentration of iodine.**

Response:	We appreciate the Editor's comment and the opportunity given to improve the quality of this paper. However, the authors would like to clarify that full characterization of iodine-doped TNTs, including the EDX and XPS elemental analysis, is presented in our recently published paper [1] (XPS: Figure 4(b) and Figure 5, Page 7-8, and EDX: Figure 6, Page 9). This statement is added in section 3.2 of the current paper. The published paper is also cited in this section. (Location: 3.2 Iodine release profile from the I-TNTs specimens)

**Reference**

[1] Taweekitikul P, Aliyu A, Decha‐Umphai D, Tantavisut S, Khamwannah J, Puncreobutr C, et al. Synthesis, characterization, and interfacial adhesion of titania iodine‐doped nanotubes architectures on additively manufactured Ti‐6Al‐4V implant. Materialwissenschaft und Werkstofftechnik. 2025;56(3):438-54.

**Response to Reviewer's Comments**

We appreciate the reviewers' careful reading of our manuscript and their thoughtful comments and critiques. The reviewers made several suggestions to improve the manuscript. Enclosed please find our step-by-step responses to address the points raised by the reviewer.

**Reviewer 1**

**Q. No. 1	While the six formulations are well-documented, the rationale for selecting specific (a) KI concentrations (0.1 and 0.5 g/L) and (b) ECA durations (210 and 240 minutes) remains unclear. The authors are encouraged to provide justification for these parameters, either from previous literature or preliminary optimization data.**

Response:	(a) The rationale for using specific KI concentrations (0.1 and 0.5 g/L)

The minimum bactericidal concentration (MBC) of povidone iodine against specific strains of *Staphylococcus aureus* and *Staphylococcus epidermidis* is a crucial parameter in evaluating its antiseptic efficacy. The previous publication reported that the minimum bactericidal concentrations (MBC) of povidone iodine against *S. aureus* and *S. epidermidis* are 1.32 g/L, and complete cytotoxicity to human cells occurs at 2 g/L [1].

Based on general knowledge, povidone-iodine solutions typically contain 1% available iodine, while KI contains 2% iodine. To achieve the same iodine content as 1.32 g/L of povidone-iodine, approximately 0.66 g/L of KI is required, ensuring that KI does not exceed 1 g/L to avoid potential cytotoxicity to human cells. In this study, we selected KI concentrations of 0.1 g/L and 0.5 g/L for preliminary analysis. We planned to increase the KI concentration up to 1 g/L if the antibacterial efficacy results were unsatisfactory.

(b) The rationale for using specific ECA durations (210 and 240 minutes)

Prior publications indicate that AR is the most influential parameter for drug release among all the dimensions of TNT. According to Miyabe et al., different lengths and diameters of nanotubes could be manipulated to control drug loading and release rates. The higher the aspect ratio, the more efficient the drug delivery system can be, as the larger surface area permits enhanced drug loading capabilities [2]. A previous study conducted by our team [3] demonstrated that ECA durations of 210 and 240 minutes resulted in the highest aspect ratio (AR) for titania nanotubes (Figure 2, Page 6 in the previous manuscript). Thus, these specific ECA durations were selected for the current investigation.

These statements were summarized and incorporated into the modified manuscript. (Location: 2.1 Preparation of iodine-supported TNT)

**Q. No. 2	A critical limitation of the study is the absence of quantitative analysis of iodine loading (e.g., via XPS or EDS). Without precise quantification of iodine content on the TNT surface, interpretation of the release kinetics remains incomplete. This limitation is briefly acknowledged in the discussion, but it significantly weakens the conclusions regarding loading efficiency and capacity.**

Response:	Thank you for this comment. The authors would like to clarify that the quantitative analysis of iodine content in the TNTs is out of the scope of this paper. However, in our recently published article [2], the approximate theoretical wt. % of iodine doped into the titania nanotubes was determined. The iodine content obtained from EDS analysis (Figure 6, Page 9) (0.05 wt. %) is found to be in agreement with the theoretical value (0.038 wt. %) (Page 5).

**Q. No. 3	The MTT assay was appropriately conducted according to ISO 10993-5 standards, and the results are clearly presented. However, the inclusion of additional cytotoxicity assays, such as Live/Dead staining or LDH assay, would further support the biocompatibility claims, particularly in the context of long-term implant-tissue interaction.**

Response:	Additional in vitro cytotoxicity assays (e.g., Live/Dead staining or LDH assay) and in vivo biocompatibility tests are planned for future investigations to support long-term implant-tissue interaction. However, the present study specifically aims to establish the perioperative cytotoxicity of this implant. Consequently, we report only the short-term relative cell viability of MC3T3-E1 cells following treatment with liquid extracts from I-TNTs samples for 24 hours and after direct exposure to the surface of I-TNTs for up to 14 days.

**Q. No. 4	Several statistical comparisons report p-values generically (e.g., "p < 0.001"). Providing exact p-values for all statistically analyzed data would improve transparency and reproducibility.**

Response:	Replace p < 0.001 with the exact p-values

1.	The cumulative iodine release profiles were significantly higher in the I-TNTs groups compared to the control group (p = 0.000).

2.	The first significant difference between I-TNT A1 and I-TNT A5 was observed at 24 hours (p = 0.014), while I-TNT B1 and I-TNT B5 showed significant differences at the final time-point of 672 hours (p = 0.000).

The exact values of the p-values were written in the manuscript as suggested.

**Q. No. 5	The FE-SEM image (page 22) successfully demonstrates the TNT structure. However, the relatively rough surface morphology of the experimental group raises concerns about stable cell attachment. It is recommended that the authors include additional FE-SEM images of the surfaces after cell culture to provide visual confirmation of cellular adhesion and morphology.**

Response:	While cell attachment is indeed an essential characteristic for orthopedic implants, it is important to note that for this specific I-TNT structure, our primary focus is on its antimicrobial properties, achieved through the controlled release of iodine. The detailed cell adhesion morphology, as observed by FE-SEM, will be presented in a subsequent study.

**Q. No. 6	The TNTs generated in this study are estimated to be approximately 6 µm in length. Longer nanotubes are generally more susceptible to delamination, especially when subjected to mechanical friction during implantation. Given the screw-type design of the implant, the authors should address potential clinical risks of delamination and propose strategies to mitigate this mechanical vulnerability.**

Response:	The potential clinical risks of delamination and proposed strategies to mitigate the effects of very long (≥ 6 µm) TNTs and implants were clearly stated and supported by the literature in the discussion section. (Location: 2^nd^ paragraph of Discussion)

**Q. No. 7	The discussion regarding iodine species (I₂, HOI, H₂OI⁺) is informative and appreciated. To further strengthen this section, the authors should consider referencing studies on iodine diffusion within nanotubular structures and the** **dominant speciation of iodine under physiological conditions.**

Response:	More information related to iodine diffusion within TNT structures was added in the discussion section (Location: 4^th^ paragraph of Discussion). The dominant speciation of iodine under physiological conditions is primarily characterized by the presence of iodide (I^−^) and iodate (IO^3−^). While I₂ and HOI can exist as transient or reactive intermediates, their equilibrium concentrations are generally very low due to rapid hydrolysis and subsequent reactions at neutral pH [4] This information was also cited as far as suggested. (Location: 4^th^ paragraph of Discussion)

**References**

1.	Van Meurs S, Gawlitta D, Heemstra K, Poolman R, Vogely H, Kruyt M. Selection of an optimal antiseptic solution for intraoperative irrigation: an in vitro study. JBJS. 2014;96(4):285-91.

2.	Miyabe S, Fujinaga Y, Tsuchiya H, Fujimoto S. TiO2 nanotubes with customized diameters for local drug delivery systems. Journal of Biomedical Materials Research Part B: Applied Biomaterials. 2024;112(7):e35445.

3.	Taweekitikul P, Aliyu A, Decha‐Umphai D, Tantavisut S, Khamwannah J, Puncreobutr C, et al. Synthesis, characterization, and interfacial adhesion of titania iodine‐doped nanotubes architectures on additively manufactured Ti‐6Al‐4V implant. Materialwissenschaft und Werkstofftechnik. 2025;56(3):438-54.

4.	Espino-Vázquez AN, Rojas-Castro FC, Fajardo-Yamamoto LM. Implications and practical applications of the chemical speciation of iodine in the biological context. Future Pharmacology. 2022;2(4):377-414.

**Reviewer 2**

**Q. No. 1	Research Background and Significance**

**The introduction clearly outlines the advantages of 3D-printed titanium alloy implants and the associated infection risks, logically proposing the innovative strategy of combining iodine with TNTs. However, some cited references (e.g., Refs. 23 and 24, published in 1975 and 1987, respectively) are outdated. It is recommended to update these with more recent studies (within the last five years) to enhance the timeliness of the discussion.**

Response:	Refs 23 and 24 were replaced with the most recent references (refs 24 and 25, respectively) as suggested.

**Q. No. 2	The study comprehensively evaluates drug release, cytotoxicity, and antibacterial activity, aligning with the standards for biomaterials research.**

**Suggestions for Improvement:**

(a) **Iodine Loading Mechanism: The "Modified ECA process" using KI is mentioned, but the binding mode of iodine ions to TNTs (e.g., physical adsorption or chemical bonding) is not explicitly described.**
(b) **In Vitro-In Vivo Correlation: The in vitro release curve shows that the MIC is reached at 7 days, but the antibacterial tests only assess 24-hour efficacy. It is recommended to supplement time-kill curves (e.g., at 1, 3, and 7 days) to clarify long-term antibacterial performance.**

Response:	(a) Binding mode of iodine ions to TNTs

The binding mode of iodine ions to titania nanotubes primarily involves chemical bonding rather than mere physical adsorption. Research indicates that iodine can form stable interactions with the titanium dioxide (TiO_2_) surface, enhancing its photocatalytic properties and antibacterial activity.

Iodine can form chemical bonds with titania, as demonstrated in iodine-doped titanium dioxide, where it is incorporated into the TiO_2_ lattice. This incorporation, involving the formation of I–O–Ti and I–O–I structures, enhances visible light absorption and photocatalytic activity [1].

In the context of carbon nanotubes, iodine can covalently bond to the surface, maintaining the electronic properties of the nanotubes. This suggests that similar covalent interactions could occur with titania nanotubes, although this is not explicitly confirmed in the provided contexts [2].

Considering the physical adsorption, it typically involves weak van der Waals forces and does not result in significant changes to the electronic structure or the crystallography of the substrate. In contrast, the literature describes scenarios where iodine not only adsorbs onto TiO_2_ nanotubes but also integrates into the lattice, modifying electronic properties and stability, which are characteristics typical of chemical bonding [3].

While some studies suggest that certain molecules can be physiosorbed onto titania nanotubes [4], the evidence for iodine indicates a preference for chemical bonding, which is crucial for its enhanced functional properties. This distinction underscores the significance of chemical interactions in enhancing the performance of titania-based materials. (Location: 3^rd^ paragraph of Discussion)

Time-kill curve

This particular implant primarily aims to prevent implant-associated infection resulting from perioperative contamination. Consequently, it adheres to the standards set by JIS Z 2801, where the antibacterial activity value is calculated based on 24-hour efficacy. Data from a time-kill curve extending up to 7 days would provide valuable insights, which we intend to present in a future study.

**Q. No. 3	Drug Release Curve (Figure 4):**

**The sustained-release characteristics are clearly described, but the release kinetics model (e.g., zero-order, first-order, or Higuchi model) is not analyzed. Fitting analysis should be added to elucidate the release mechanism.**

Response:	The Higuchi model most accurately describes the observed release profile. This profile exhibited an initial burst release, likely due to surface-adsorbed iodine, succeeded by a slower, diffusion-controlled release from the I-TNT matrix. The cumulative release gradually increased following a square root of time behavior, as opposed to a linear progression.

**Q. No. 4	Formatting and Language**

**Ensure consistent formatting of references.**

**Strengthen the discussion section by enhancing mechanistic explanations and linking findings to clinical significance.**

**Link findings to clinical significance.**

Response: The manuscript is formatted according to the journal's guidelines. The manuscript was further proofread. The discussion section was strengthened by adding the potential clinical risks of TNT delamination and proposing strategies to mitigate its effects, especially when TNT is too long (≥ 6 µm).

**References**

1. He Z, Yu Y, Wang D, Tang J, Chen J, Song S. Photocatalytic reduction of carbon dioxide using iodine-doped titanium dioxide with high exposed {001} facets under visible light. RSC Advances. 2016;6(28):23134-40.
2. Coleman KS, Chakraborty AK, Bailey SR, Sloan J, Alexander M. Iodination of single-walled carbon nanotubes. Chemistry of materials. 2007;19(5):1076-81.
3. Yang X, Chen N-F, Huang X-L, Lin S, Chen Q-Q, Wang W-M, et al. Iodine-doped TiO2 nanotube coatings: a technique for enhancing the antimicrobial properties of titanium surfaces against Staphylococcus aureus. Journal of Orthopaedic Surgery and Research. 2023;18(1):854.
4. Chen Q, Jia Y, Liu S, Mogilevsky G, Kleinhammes A, Wu Y. Molecules immobilization in titania nanotubes: a solid-state NMR and computational chemistry study. The Journal of Physical Chemistry C. 2008 Nov 6;112(44):17331-5.
