# Supplementary material for: Biocompatibility and antimicrobial efficacy of iodine-supported titania nanotubes on 3D-printed Ti-6Al-4V implants
Source: PLoS One. 2025 Dec 26;20(12):e0339618. doi: 10.1371/journal.pone.0339618 (PMC12742766; doi:10.1371/journal.pone.0339618)
Supplement: Attachment — Submitted filename: Response to Reviewer 2nd revision Final 011225.docx [file pone.0339618.s006.docx]

**Response to Reviewer #3’s Comments**

**1. The authors characterize the iodine release profile from I-TNT samples in Section 3.2. Statistical tests on the cumulative iodine release at different time points were performed. A more rigorous approach to characterize release profiles would be to fit kinetic models and compare parameters, rather than the present approach. A prior reviewer suggested that the authors perform kinetic modeling, and the authors have responded that “The Higuchi model most accurately describes the observed release profile…”, but no fitting analysis is found in the manuscript or supporting information. Some kinetic modeling may enhance the manuscript’s discussion of the iodine release mechanism via diffusion through TNT and may offer predictions for long term, in vivo antimicrobial activity.**

**Response:** The preliminary fitting analysis was performed using the following sequential steps:

1. As previously stated, a limitation of this study was the quantification of the total iodine content directly on the implant surface. Consequently, we relied on the theoretical iodine loading within the titania nanotubes to define 100% release. To achieve this, the total theoretical iodine content of the I-TNT samples was first calculated. Based on the established formulation [1], the theoretical iodine content was 0.038 wt.-%. Using the average weight of the I-TNT samples (768.53 ± 20.71 mg (detailed in the *minimal data set*), the total theoretical iodine content was calculated to be

$$\frac{0.038x768.53x1000}{100}=292.04 \mu g$$

2. The percentage of cumulative iodine release at each sampling point was subsequently calculated by normalizing the measured iodine content to the total theoretical iodine content.

3. To identify the release kinetics, the experimental data were fitted to several key kinetic models, including the Zero-order, First-order, Higuchi, Hixson-Crowell, and Korsmeyer–Peppas models. The best-fit model was determined by comparing the correlation coefficient (R^2^) values. This fitting analysis was performed using Microsoft Excel.

The kinetic analysis was performed using the iodine release profiles obtained from all four I-TNT formulations. Upon comparison of the correlation coefficients (R^2^), **the Higuchi model** was identified as the best statistical fit for the experimental data (detailed results are provided in **Supplement 3**). This result strongly suggests that the drug release mechanism is consistent with the principles of the Higuchi model.

The adherence of the release data to the Higuchi model implies a **diffusion-controlled sustained-release profile**. This model is fundamentally based on the principle that the drug release rate is proportional to the square root of time (√*t*). Mechanistically, this demonstrates that the primary governing process is Fickian diffusion. Iodine molecules move out of the I-TNT matrix driven by a concentration gradient, passing through pores formed as the surrounding medium penetrates the TNT. This characteristic of a diffusion-controlled sustained release is particularly advantageous for controlled-release formulations, enabling more predictable dosing and subsequently reducing the risk of concentration-related side effects associated with plasma concentration peaks.

**Reference:**

[1] Taweekitikul P, Aliyu A, Decha‐Umphai D, Tantavisut S, Khamwannah J, Puncreobutr C, et al. Synthesis, characterization, and interfacial adhesion of titania iodine‐doped nanotubes architectures on additively manufactured Ti‐6Al‐4V implant. Materwiss Werksttech. 2025;56(3):438-54.

**2.The antimicrobial data is presented in Tables 3 and 4. Certain conditions are quite effective and results in measurements of “<4.55” which this reviewer assumes to the be limit of detection for this study and specific assay. For transparency, the authors should document the dilution conditions and any other relevant data used to determine this limit of detection.**

**Response:** The quantification of viable bacteria (CFU/cm^2^) (equation 1)

$$N=\frac{CxDxV}{A}(1)$$

where	N: number of viable bacteria (per 1 cm^2^ of test piece)

C: count of colonies (average count of colonies of two petri dishes adopted)

D: dilution factor (that of dilution dispensed into petri dishes adopted)

V: volume of SCDLP broth used for washout (mL)

A: surface area of test piece that was exposed to bacteria (cm^2^)

**C = 1** (when the count of colonies C is “<1”, C is taken as “1”), **D = 1**, **V = 10** ml, **A = 2.2** cm^2^.

N shall be expressed as “**<4.55**”.

**3.For Fig. 5, 6, 7 and Tables 3 and 4, the sample naming convention seems to have changed. Is “TNT-A” the sample described as “I-TNT A1” in Table 1?**

**Response:** The “TNT-A” in Fig. 5, 6, 7 and Tables 3 and 4 is labelled correctly.

For clarity across the results, the sample designation 'TNT-A' refers specifically to the TNT synthesized using the Electrochemical Anodization (ECA) technique. As detailed in Section 2.1 ('Preparation of iodine-supported TNT') and illustrated in Figure 1, the synthesis parameters for TNT-A involved a constant applied voltage of 60 V and a duration of 210 minutes. This specific set of parameters was selected because TNT-A represents the TNT sample exhibiting the highest aspect ratio identified in our previous publication [1]

The data presented in Figures 5, 6, and 7 and Tables 3 and 4 facilitate the comparison of TNT-A (TNT samples without iodine support) against the functionalized samples, I-TNT A5 and I-TNT B5 (iodine-supported TNT samples), specifically focusing on their respective cytotoxicity and antibacterial efficacy.

**Reference:**

[1] Taweekitikul P, Aliyu A, Decha‐Umphai D, Tantavisut S, Khamwannah J, Puncreobutr C, et al. Synthesis, characterization, and interfacial adhesion of titania iodine‐doped nanotubes architectures on additively manufactured Ti‐6Al‐4V implant. Materwiss Werksttech. 2025;56(3):438-54.
